# 141. Impact of Diagnostic Stewardship of Respiratory Cultures in the Pediatric ICU

**DOI:** 10.1093/ofid/ofad500.214

**Published:** 2023-11-27

**Authors:** Christine D Hamilton, Julia Fabricio, William Wilson, Isabelle Riley, Jenny Boyd, Michael Swartwood, Casey Olm-Shipman, Zachary Willis

**Affiliations:** Vanderbilt University Medical Center, Nashville, Tennessee; University of North Carolina at Chapel Hill Eshelman School of Pharmacy, Chapel Hill, North Carolina; UNC Medical Center, Raleigh, NC; University of North Carolina at Chapel Hill School of Medicine, Durham, North Carolina; University of North Carolina at Chapel Hill School of Medicine, Durham, North Carolina; University of North Carolina Medical Center, Chapel Hill, North Carolina; University of North Carolina at Chapel Hill School of Medicine, Durham, North Carolina; University of North Carolina School of Medicine, Chapel Hill, NC

## Abstract

**Background:**

Lower respiratory cultures (LRC) obtained from respiratory devices in intensive care units (ICUs) have poor specificity and positive predictive value (PPV). False-positive LRC are common and can lead to unnecessary antimicrobial use; therefore, LRC in ICUs are an ideal target for diagnostic stewardship. Our Quality Improvement (QI) project aimed to reduce use of LRC in patients not meeting guideline criteria in a pediatric ICU (PICU).

**Methods:**

Criteria for respiratory culture collection in the PICU, including systemic signs of illness plus respiratory symptoms (Figure 1), were developed through a multidisciplinary process using best evidence-based practice. These guidelines were introduced to PICU providers with a start date of 12/1/2021. Every respiratory culture sent in the PICU was audited for guideline concordance during the 3 months prior to and 13 months following implementation. Periodic feedback and reminders were provided to PICU providers.

Figure 1

**Figure:**
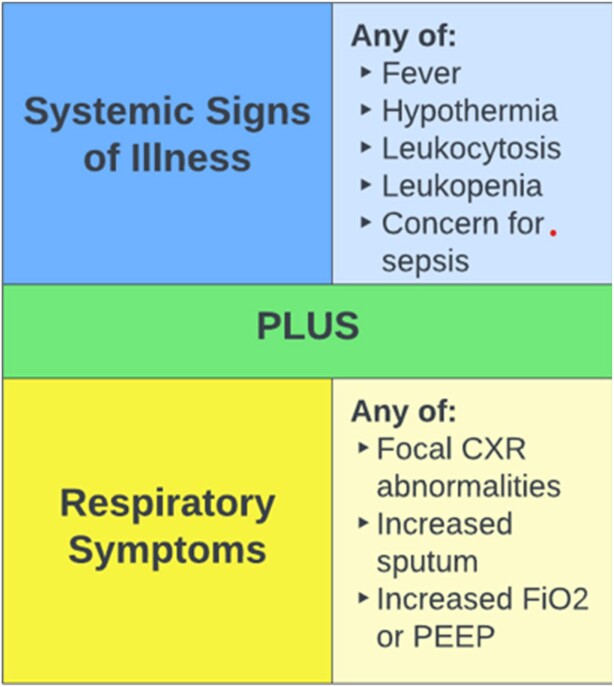

Criteria for guideline-concordant respiratory cultures.

**Results:**

Over a 16-month period, we audited 239 respiratory cultures from 145 patients admitted to our hospital's PICU. Prior to the introduction of the diagnostic guidelines, 23 of 47 (48.9%) LRC were retrospectively deemed guideline discordant. Following guideline introduction, 40 of 192 LRC were guideline-discordant (20.8%), representing a 57% reduction in guideline-discordant cultures (Figure 2). The total number of guideline-concordant LRC was 8.0/month pre- and 11.7/month post-implementation, and the proportion that were positive did not change (41.7% pre- *vs* 42.1% post-implementation). Guideline-discordant LRC that grew a potential pathogen were more frequent pre-implementation (Figure 3). Fewer patients received antibiotics attributable to guideline-discordant LRC following implementation (5.3 *vs* 1.6 patients per month, Figure 4).

Table 1

**Figure:**
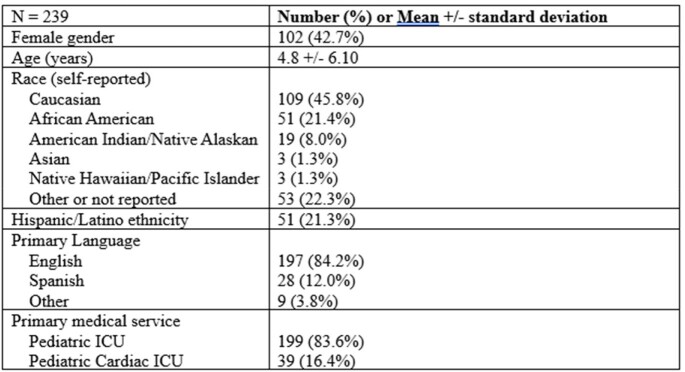

Patient characteristics, by case.

Figure 2

**Figure:**
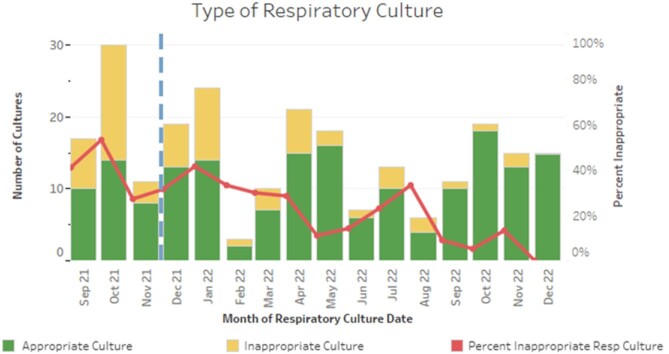

Proportion of lower respiratory cultures that were guideline-concordant (appropriate) per month. Red line: Proportion of respiratory cultures that were guideline-discordant.

Figure 3

**Figure:**
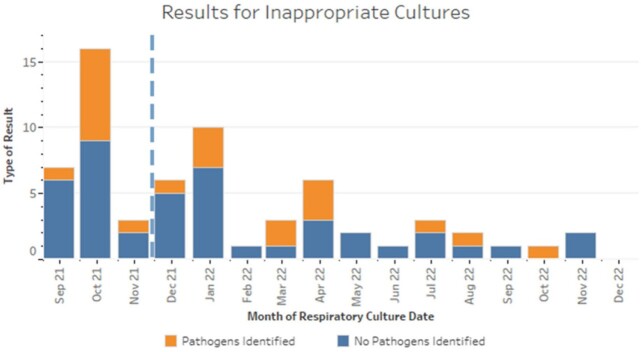

Guideline-discordant (inappropriate) cultures that resulted positive or negative for pathogens, by month. Orange: Guideline-discordant cultures with pathogens identified.

**Conclusion:**

In our study, a diagnostic stewardship intervention reduced use of LRC in patients with low pre-test probability, with a reduction in antibiotic courses directed at such cultures. Utilization of guideline-concordant respiratory cultures did not change following guideline implementation. Diagnostic stewardship can reduce the frequency of LRC in patients with low pre-test probability for pneumonia, with substantial antimicrobial stewardship benefits.

Figure 4

**Figure:**
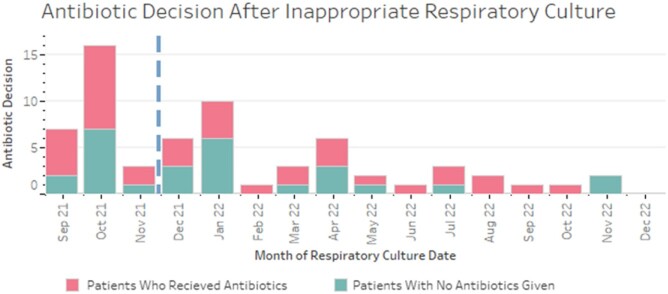

Number of patients with a guideline-discordant (inappropriate) respiratory culture who received antibiotics attributable to the culture. Pink: received antibiotics after guideline-discordant culture.

**Disclosures:**

**Zachary Willis, MD, MPH**, Merck Sharp & Dohme Corp: Grant/Research Support|Pfizer Inc: Grant/Research Support

